# Tumor targeting of innate and adaptive immunity by the adoptive cell transfer of engineered T lymphocytes co-expressing iNKT and tumor-specific MHC-I TCRs

**DOI:** 10.1186/2051-1426-2-S3-P11

**Published:** 2014-11-06

**Authors:** Benjamin O Tschumi, Justyna Iwaszkiewicz, Lianjun Zhang, Stéphanie Corgnac, Jean-Pierre Mach, Pedro Romero, Alena Donda

**Affiliations:** 1 University of Lausanne, Epalinges, Switzerland; 2 Swiss Institute of Bioinformatics, Lausanne, Switzerland; 3 Ludwig Center for Cancer Research, Zurich, Switzerland

## 

CD1d-restricted invariant NKT cells (iNKT) exert potent anti-tumor effects by virtue of their ability to transactivate NK cells, dendritic cells and T lymphocytes. However, their use in cancer immunotherapy has been limited by their short-lived activation followed by a phase of long-term anergy after a single injection of the high affinity CD1d ligand alpha-galactosylceramide (αGC). Instead, we have demonstrated that repeated injections of recombinant soluble αGC-loaded CD1d molecules resulted in the sustained iNKT and NK cell activation, which correlated with prolonged antitumor effects when the αGC/sCD1d was fused to an anti-tumor scFv fragment. In addition, we recently showed that αGC/CD1d-antitumor fusion protein greatly increased the efficacy of a therapeutic peptide/CpG-based cancer vaccine, first as an adjuvant during T cell priming and second, as a therapeutic agent to redirect immune responses to the tumor site.

To optimize the synergy between iNKT cells and cytotoxic T lymphocytes (CTLs), we aim at conferring both antigen specificities to the same T lymphocyte by transducing iNKT cells with high avidity MHC-I-restricted TCR, or conversely transduce CTLs with the CD1d-restricted iNKT invTCR. Indeed, the simultaneous triggering of transduced HLA-A2/NY-ESO-I TCR and of the endogenous iNKT TCR led to increased cytokine secretion and killing of HLA-A2 and HER2 positive tumor cells, when pulsed with the antigenic peptide and coated with the CD1d-anti-HER2 fusion protein. To reduce TCR mispairing between endogenous and transduced TCRs, we are developing human and mouse single chain iNKT TCRs (iNKT scTv) fused to CAR-derived activation domains. The stability between the murine Va and Vb variable domains of the iNKT scTv is being optimized by site-directed mutagenesis and by spacer design. The resulting variants transduced in MHC-I-restricted T cells are tested for their binding to αGC/CD1d multimer and for TCR function. In vivo studies will involve the adoptive transfer of iNKT scTv-transduced tumor-specific CTLs in immunized mice grafted with tumor cells co-expressing the MHC-I-restricted and CD1d-targeted antigens.

It is expected that this approach will confer CD1d-glycolipid specificity to tumor-specific CD8 T cells, in which a major advantage is the availability of a single invariant TCR that can be offered to all patients independently of their MHC-I haplotype.

